# Gamma radiation-induced synthesis of novel PVA/Ag/CaTiO_3_ nanocomposite film for flexible optoelectronics

**DOI:** 10.1038/s41598-023-38829-9

**Published:** 2023-07-31

**Authors:** M. I. A. Abdel Maksoud, Soraya Abdelhaleem, Eman K. Tawfik, A. S. Awed

**Affiliations:** 1 Radiation Physics Department, National Center for Radiation Research and Technology (NCRRT), Egyptian Atomic Energy Authority (EAEA), Cairo, Egypt; 2 Higher Institute for Engineering and Technology at Manzala, El Manzala, Egypt

**Keywords:** Optics and photonics, Physics, Materials science, Materials for devices, Materials for optics

## Abstract

A flexible nanocomposite film based on polyvinyl alcohol (PVA), silver nanoparticles, and calcium titanate (CaTiO_3_) was synthesized using gamma radiation induced-reduction. Temperature-dependent structural, optical, DC electrical conductivity, electric modulus, and dielectric properties of PVA/Ag/CaTiO_3_ nanocomposite film were investigated. The XRD pattern proved the successful preparation of the nanocomposite film. Also, as the temperature increases, the average crystallite sizes of CaTiO_3_ and Ag nanoparticles decrease from 19.8 to 9.7 nm and 25 to 14.8 nm, respectively. Further, the optical band gap increased from 5.75 to 5.84 eV with increasing temperature. The thermal stability is improved, and the semiconductor behavior for PVA/Ag/CaTiO_3_ nanocomposite film is confirmed by thermal activation energy ΔE with values in the 0.11–0.8 eV range. Furthermore, the maximum barrier W_m_ value was found of 0.29 eV. PVA/Ag/CaTiO_3_ nanocomposite film exhibits a semicircular arc originating from the material’s grain boundary contributions for all temperatures. The optical, DC electrical conductivity, and dielectric properties of the PVA/Ag/CaTiO_3_ nanocomposite film can be suitable for flexible electronic devices such as electronic chips, optoelectronics, and energy storage applications.

## Introduction

It is well known that mixing polymers and nanomaterials generates unusual molecular interactions essential in improving the overall system properties^1^. Polymer nanocomposites have attracted great interest due to their unique properties, allowing them to be used in specific applications such as energy storage and optoelectronic devices. Usually, polymers are utilized as a host material for nanoparticles NPs. Adding NPs into the polymer matrix improves the properties of the polymer, as they significantly improve the characteristics of the polymer nanocomposites when compared to the pristine polymer, owing to their high surface-to-volume ratio^2^.

Polyvinyl alcohol (PVA) has emerged as one of the most efficient and widely used polymeric materials; it has been utilized in numerous technological applications such as encapsulation of photovoltaic devices, sensors, electronic coatings for noise reduction, drug delivery systems, and reinforcement fibers in cement, etc. The wide range of applications of PVA is because of its remarkable properties such as low cost, good film-forming ability, high tensile strength, flexibility, excellent chemical resistance, and water solubility^3,4^. Due to its ease of processing and chemical stability, PVA is widely used to fabricate various polymer composites^5–8^.

Recently, perovskite materials with a general formula ABO_3_ have attracted significant research interest. Owing to their diverse physical properties, such as structural flexibility, tunable band gap, low-cost production, electron mobility, and high thermal stability^9^, perovskite materials have been widely used in several applications such as photovoltaic devices, batteries, photodetectors, sensing devices, light emitting diodes, fuel cells, and photocatalysis^4^. Many previous studies have highlighted the properties and applications of several perovskite materials such as SrZrO_3_, SrRuO_3_, CaGeO_3,_ PbTiO_3_, SrTiO_3,_ BaTiO_3_, GdFeO_3_ and CaTiO_3_^10,11^. Among these perovskites, Calcium titanate (CaTiO_3_), has gained much interest due to its remarkable properties of optoelectronic, ferroelectricity, and photocatalytic activity^12^. Calcium titanate CaTiO_3_ is an n-type semiconductor^13^ with a perovskite structure; it possesses excellent characteristics, such as earth-abundance and non-toxicity of its constituent elements, cost-effective, high dielectric constant, ease of synthesis, and high chemical stability^14^. Several methods have been reported to prepare CaTiO_3_, such as solid-state reaction^15^, co-precipitation^16^, mechano-chemical milling, sol–gel^17^, and hydrothermal process^18^.

CaTiO_3_ has three phases: orthorhombic, tetragonal, or cubic structure. The cubic structure is stable at a temperature higher than 1374 °C, the tetragonal structure is stable at temperatures 1250–1350 °C, and the orthorhombic structure is stable at a temperature lower than 1213 °C. Owing to these excellent properties, CaTiO_3_ has been considered a promising candidate to be combined with PVA to form a polymer nanocomposite with a wide range of applications^3,4^. Several studies have reported that loading nanosized metal to CaTiO_3_ improves its optical, electrical, and photocatalytic properties and stabilizes the composite. The addition of silver nanoparticles (Ag NPs) to CaTiO_3_ has shown improved performance for the overall composite^19^.

The radiation procedure for nanoparticle production is remarkably simple; it typically involves irradiating aqueous solutions holding appropriate precursors with gamma rays or accelerated electrons at room temperature. In many cases, there is not much difference in the quality of nanoparticles generated via gamma or accelerated electron irradiation. Depending on the intended use, the nanoscale solid phase or colloidal nanoparticles generated in a radiation environment may be subjected to further processing steps. Certain enhanced forms of the radiation technique also permit the rapid creation of massive quantities of powder materials at the micro or nano size, making them suitable for large-scale industrial scales^20^. With advantages like using non- or low-toxic precursors, non-toxic solvents, low reaction waste product growth, low dangerous waste generation, and few chemical reagents, radiation-induced processes, especially those that exploit the interaction of ionizing radiation with solvents, might offer a unique opportunity of competing with conventional processes for achieving essential objectives in a synthesis process^21^.

Generally, the Ag NPs are short-lived in an aqueous solution because they agglomerate quickly. Many research studies have gamma radiation to reduce the silver ions^22^. Among the several synthesizing techniques of Ag NPs, the reduction of Ag ions by gamma radiation has shown some advantages, such as the full reduction of metal precursors, high stability, and being environmentally safe^23,24^. In our previous work, we reported the gamma radiation induced-synthesis of Ag/NiMn_2_O_4_ for energy storage^25^, chitosan/Ag/Mn-Mg ferrite composite for plant growth^26^, and Ag/MoS_2_/ZnCo_2_O_4_ for wastewater treatment applications^27^. In this work, we reported, for the first time, the synthesis of a novel PVA/Ag/CaTiO_3_ nanocomposite by gamma irradiation. Afterward, we investigated the effect of temperature on structural, optical, thermal, DC electrical conductivity and dielectric properties of the polymer nanocomposite.

## Materials and methods

The polyvinyl alcohol (PVA) with Mw = 125,000 gm/mole, calcium carbonate (CaCO_3_), titanium dioxide (TiO_2_) were procured from Sigma-Aldrich, isopropanol, and silver nitrate (AgNO_3_) were purchased from Merck.

### Preparation of calcium titanate nanopowder

Calcium titanate powder was prepared from calcium carbonate (CaCO_3_) and titanium dioxide (TiO_2_) by solid-state reactions method. The raw materials were weighed by the stoichiometric 1:1 (Ca: Ti) molar ratio. Then, the powder was ground by pestle and mortar for 30 min and in a ball milling for 8 h. After the homogenization, mixtures were sintered in an air-resistive furnace at temperatures of 1000 °C for 2 h with a heating rate of 10 °C/h. Finally, the powder was  manually crushed with a pestle and mortar to obtain a uniform CaTiO_3_ powder.

### Fabrication of PVA/Ag/calcium titanate nanocomposite

0.5 g of AgNO_3_ was added to 2 g of CaTiO_3_ and 10 ml of isopropanol and stirred for 60 min using magnetic stirring. Then, the examined samples' solutions were irradiated with 50 kGy (dose rate of 0.8 kGy/h) at ambient conditions^28^. The radiation process was achieved using Co-60 gamma-cell sources^29–31^. As presented in previous work^26,32,33^, water radiolysis induced by gamma rays in an aqueous Ag^+^ solution results in the generation of a significant number of highly effective reducing (hydrated electrons (e^−^_aq_), hydrogen atoms (H⋅), and oxidizing radicals as HO⋅. Ag NPs are formed when silver ions are converted to silver atoms, which serve as individual nucleation centers, and subsequent coalescence forms Ag NPs. Isopropyl alcohol (isopropanol) may be used to scavenge the ⋅OH produced by radiation.

**Figure 1:**
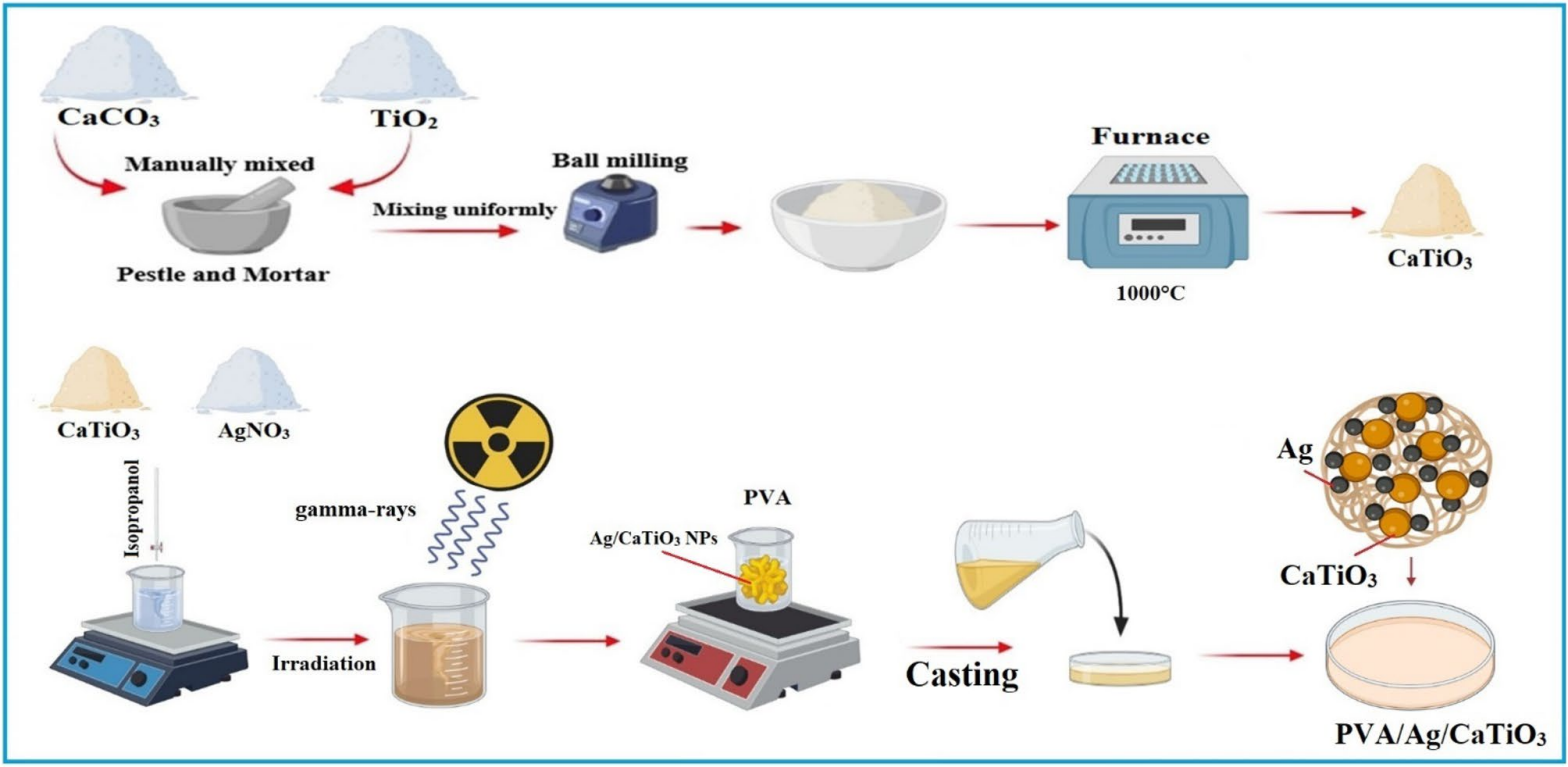
Schematic representation of the PVA/Ag/CaTiO_3_ nanocomposite film synthesis.

A solution of 6 wt% PVA was made by dissolving 6 g of PVA in 100 ml of deionized water at 70 °C. while stirring the mixture constantly until a homogenous solution had been achieved. After that, a specified concentration of Ag/CaTiO_3_ was mixed with PVA solution for 2 h during stirring. The thick solution was cast onto a transparent glass plate using a film applicator and left to dry. After one week of drying in air at room temperature, the PVA/Ag/CaTiO_3_ nanocomposite was scraped off of the glass plate, see Fig. 1. After that, the PVA/Ag/CaTiO_3_ nanocomposite films were exposed to different temperatures (313, 323, 333, 343, 353, 363, and 373 K) for 30 min.

### Characterization of PVA/Ag/CaTiO_3_ nanocomposite

The crystal structure and phase analysis of pure CaTiO_3_, Ag/CaTiO_3_ NPs, and PVA/Ag/CaTiO_3_ nanocomposite were characterized via XRD Shimadzu 6000 with 40 kV and 30 mA and scanning rate of 8°/min as operating conditions. Fourier transforms infrared (FT-IR) spectroscopy has been employed to identify the functional groups in CaTiO_3_ powder and PVA/Ag/CaTiO_3_ nanocomposite films (NICOLET iS10 model instrument). Moreover, high-resolution transmission electron microscopy (HR-TEM, (JEOL-JEM-100 CX)) was used to provide sufficient information on the particle size and the selected area electron diffraction (SAED) pattern of Ag/CaTiO_3_ NPs. A scanning electron microscope (SEM, (JEOL JSM-5600 LV, Japan)), at variable vacuum without any coating at 12 kV accelerating voltage with a back-scatter detector, was also employed to develop surface images of PVA/Ag/CaTiO_3_ nanocomposite to provide a clear insight into the morphology of the PVA/Ag/CaTiO_3_ nanocomposite film surface. The energy-dispersive X-ray analysis spectra were also used to acquire the elemental composition and mapping pictures (EDX, JEOL JSM-5600 LV, Japan.). Also, TGA-50 Shimadzu, with a heating rate of 10°/min in an N_2_ environment in the temperature range 293–873 K, was used to address the thermal stability of the PVA/Ag/CaTiO_3_ nanocomposite. Using a UV–vis-NIR spectrophotometer (Jasco, V-570), we conducted the optical characteristics from 230 to 1100 nm. As electrodes for electrical testing, silver paste is pasted to both parallel edges of samples with dimensions of (1 cm × 1 cm) and a thickness of 0.7 mm. For AC tests in the frequency range of 100 Hz–5 MHz, a programmable automated RLC bridge, model Hioki 3532 Hitester, has been employed. A k-type thermocouple has been used to monitor the sample's temperature.

## Results and discussion

### Structural analyses

**Figure 2:**
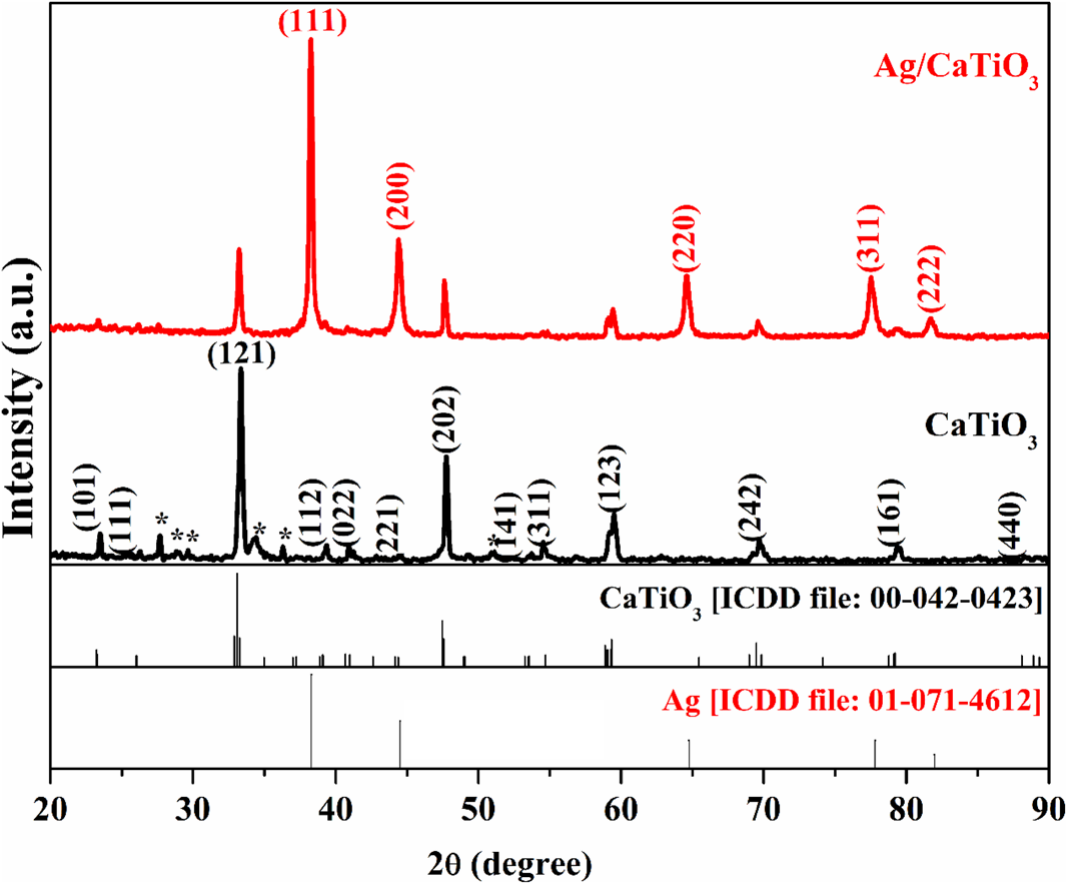
XRD diffraction patterns of CaTiO_3_ and Ag/CaTiO_3_ NPs.

Figure 2 shows XRD Diffraction patterns for pure CaTiO_3_ (CTO) nanoparticles and Ag/CaTiO_3_ nanocomposite. The diffraction pattern of pristine CaTiO_3_ shows peaks that correspond to (101), (111), (121), (112), (022), (221), (202), (141), (311), (123), (242), (161), and (440) diffraction planes of CaTiO_3_ [ICDD file: 00-042-0423]. These observed peaks correspond to orthorhombic symmetry with a perovskite-like structure^14,34^. Minor secondary phases were observed in the XRD pattern, which belongs to TiO_2,_ Ca(OH)_2,_ and CaO phases^35,36^. The average crystallite size of CaTiO_3_ was estimated using WinFit 1.2.1 (1997) software^37^, which was approximately 24.5 nm.

The diffraction pattern of Ag/CaTiO_3_ shows the characteristic diffraction peaks of Ag nanoparticles NPs, which correspond to (111), (200), (220), (311), (222) planes of the cubic structure of Ag [ICDD file: 01-071-4612], indicating that the Ag NPs were successfully loaded on the surface of CaTiO_3_^38^. The highly intense peaks of Ag NPs are due to the high amount of free silver in the composite^39^. A slight shift in the diffraction peaks of CaTiO_3_ toward lower angles was observed; this could be attributed to the internal stress arising from the loading of Ag NPs to the lattice^40^. The broadening of the CaTiO_3_ NPs peaks is decreased after Ag NPs loading, which increases the average size of CaTiO_3_ NPs to 26.9 nm. The sharp diffraction peaks in both patterns of CaTiO_3_ NPs and Ag/CaTiO_3_ NPs indicate the high crystalline nature of these samples^41^.

**Figure 3:**
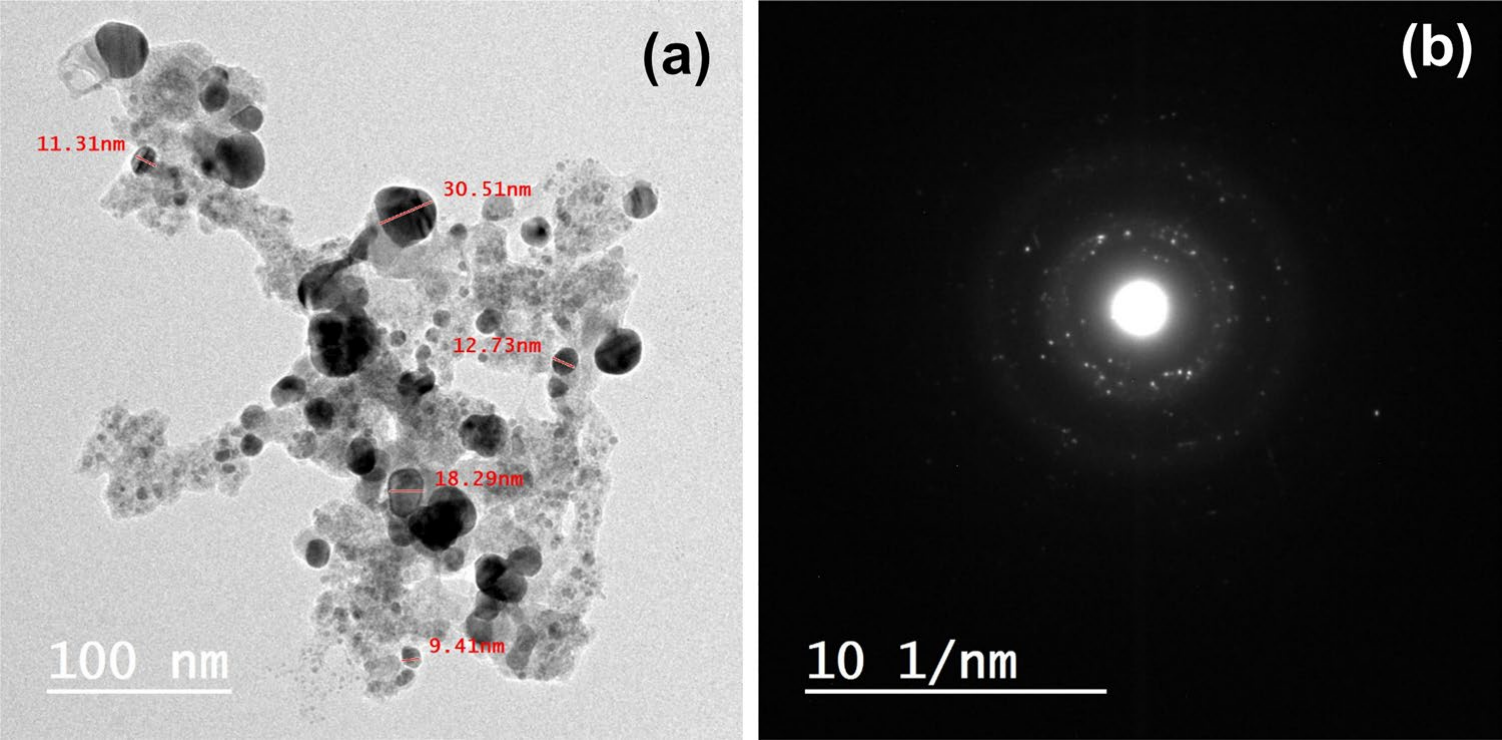
(**a**) TEM image and (**b**) SAED pattern of Ag/CaTiO_3_ NPs.

Figure 3a shows a transmission electron microscope (TEM) image of Ag/CaTiO_3_ NPs. This figure demonstrates that Ag/CaTiO_3_ NPs are spherical NPs with a particle size between 11 and 30 nm. These results are consistent with the XRD calculations. The selected area electron diffraction (SAED) pattern of Ag/CaTiO_3_ NPs, Fig. 3b, reveals bright spots at regular positions, which is evidence that the Ag/CaTiO_3_ NPs have a crystalline structure.

**Figure 4:**
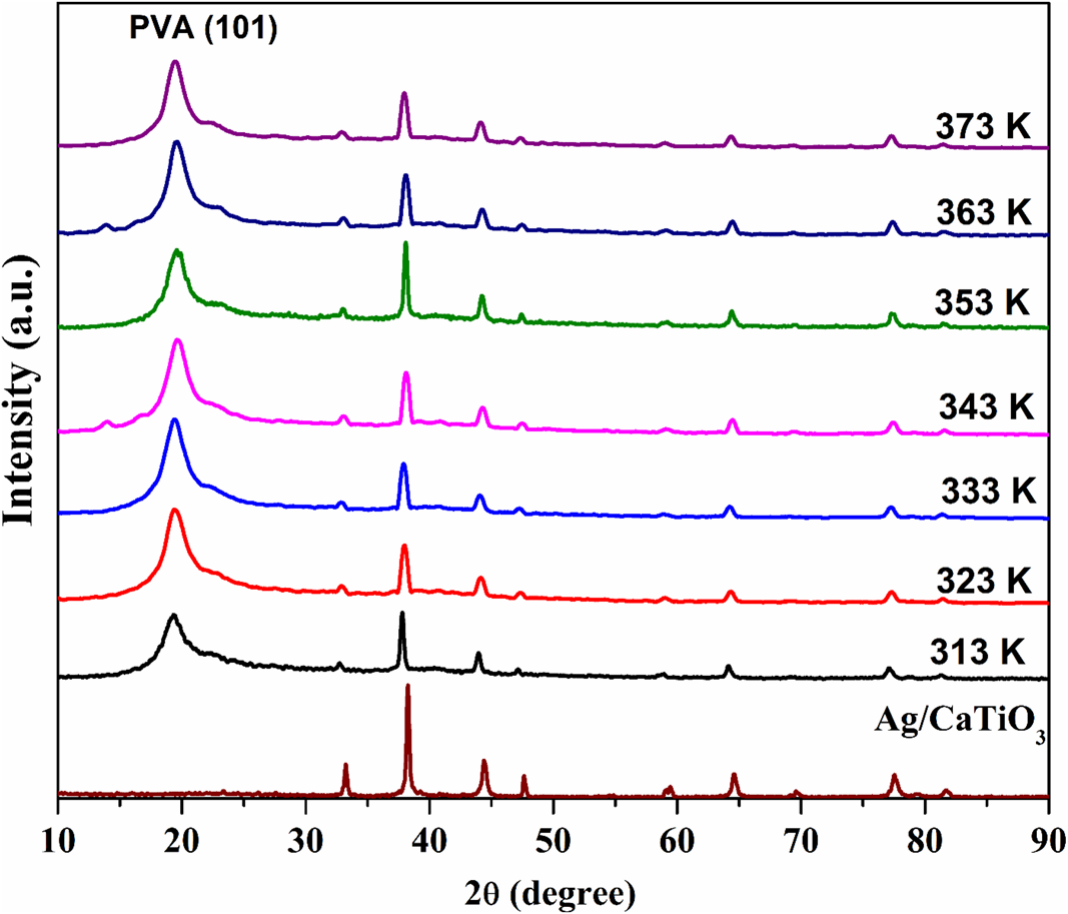
XRD patterns of PVA/Ag/CaTiO_3_ nanocomposite at different temperatures.

Figure 4 shows XRD diffraction patterns of PVA/Ag/CaTiO_3_ nanocomposite at different temperatures (313, 323, 333, 343, 353, 363, and 373 K). The typical diffraction peak of PVA, which corresponds to the (101) plane, appeared along with the diffraction peaks of Ag/CaTiO_3_ NPs. It is observed that the intensities of the diffraction peaks of Ag/CaTiO_3_ NPs are severely decreased after the dispersion of the nanoparticles into the polymer matrix due to the higher content of PVA amorphous polymer, which reduces the crystallinity^42^. The average crystallite size for the most intense peak of CaTiO_3_ NPs decreases from 19.8 nm at 313 K to 9.7 nm at 373 K. Furthermore, the diffraction peaks of Ag/CaTiO_3_ NPs are shifted toward smaller angles after dispersing in the PVA matrix, indicating the intercalated structure owing to the formation of PVA/Ag/CaTiO_3_ nanocomposite^43,44^. As the temperature increases from 313 to 373 K, the FWHM of the diffraction peaks increases, and then the average crystallite size decreases from 25 to 14.8 nm for the intense peak of Ag NPs. However, at 353 K, the intensity of the diffraction peaks is increased, and the peaks become narrower; the FWHM decreased to 1.33, 0.36, and 0.31° for the most intense peaks of PVA, CaTiO_3_, and Ag, respectively, indicating improved crystallinity. This may be attributed to the glass transition temperature of PVA, at which the physical properties of a polymer nanocomposite change^45–47^.

**Figure 5:**
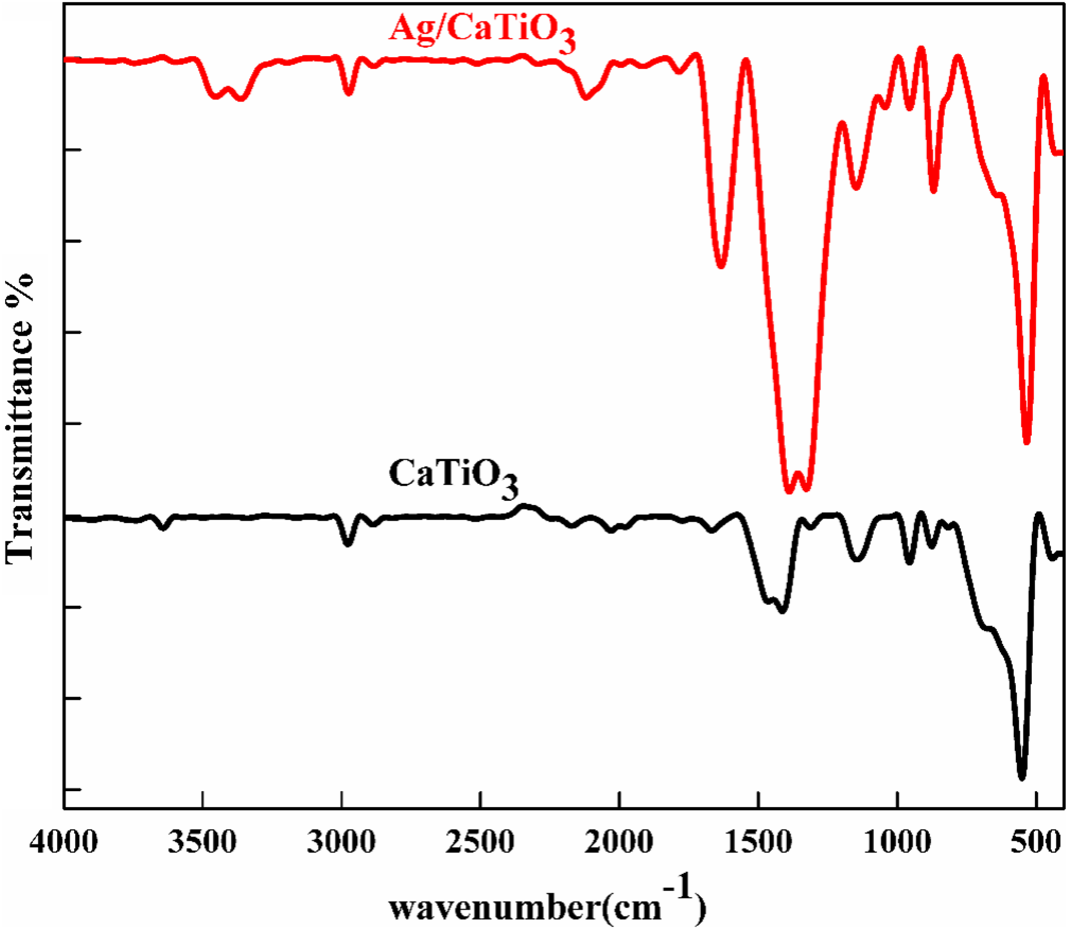
FTIR spectra of pure CaTiO_3_ and Ag/CaTiO_3_ NPs.

Figure 5 shows FTIR spectra of pure CaTiO_3_ and Ag/CaTiO_3_ NPs. The spectra show two strong bands at 3400 cm^–1^ and 3200 cm^−1^ that corresponded to vibration stretching in the O–H group that is suitable for the existence of hydroxyl group O–H, and the peak at 1721 cm^−1^ is attributed to the stretching vibrations of carbonyl (C=O) groups^48^. The bands at 1615 and 1375 cm^−1^ arise due to the stretching vibrations of C=C and bending vibrations of the carboxyl (C–OH) group. It’s noticed that the two characteristic peaks at 534 and 434 cm^−1^ in the spectrum of CaTiO_3_ NPs are related to the stretching and bending vibrations of Ti–O bonds^49^.

It can be observed that pure CaTiO_3_ and Ag/CaTiO_3_ NPs exhibited essential differences in the intensity of some peaks. Due to the effect of gamma irradiation, the intensities of peaks at 3440 and 1650 cm^−1^ of Ag/CaTiO_3_ NPs significantly increased when compared to that of pure CaTiO_3_ NPs, and there is a shift in some peaks to lower wavenumber in the spectrum of Ag/CaTiO_3_ NPs which corresponding to bonding interaction of Ag and CaTiO_3_ NPs which confirms the successful formation of Ag/CaTiO_3_ NPs^50^.

**Figure 6:**
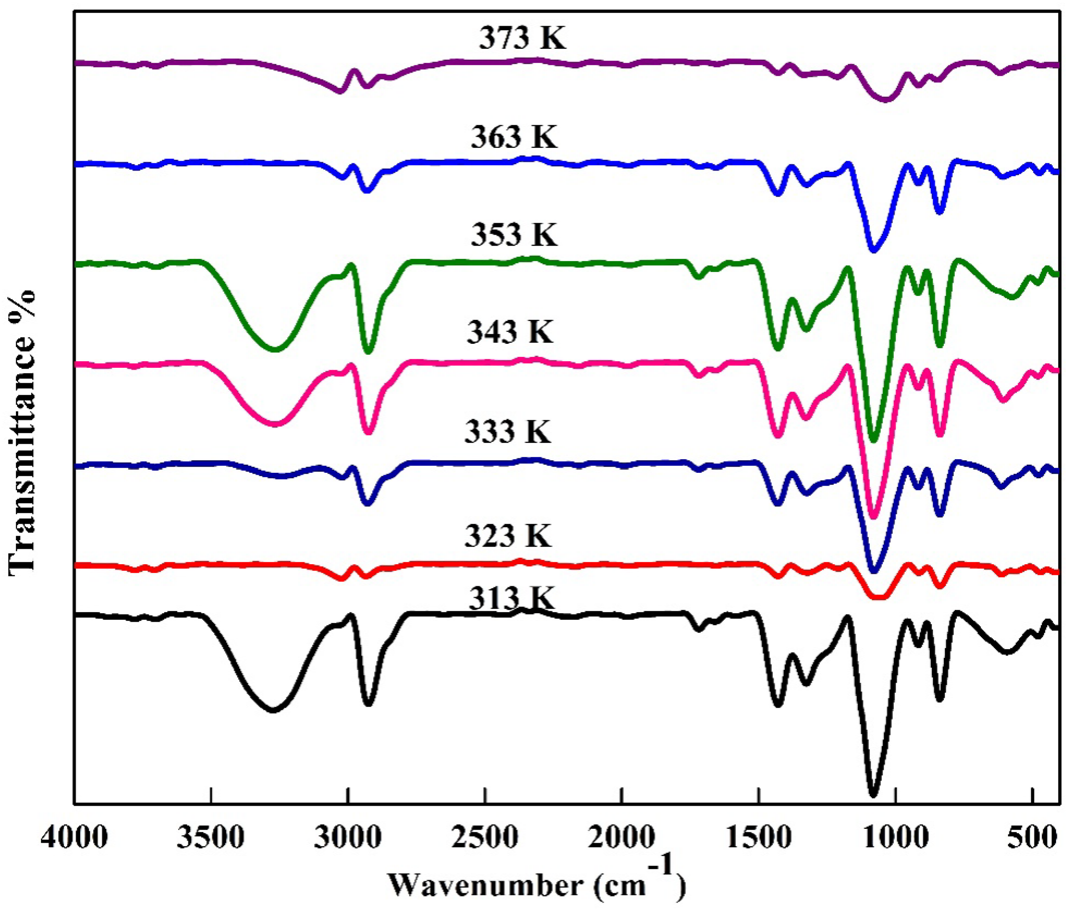
FTIR spectra of PVA/Ag/CaTiO_3_ nanocomposite with different temperatures (313, 323, 333, 343, 353, 363, and 373 K).

Figure 6 shows the FTIR spectra of PVA/Ag/CaTiO_3_ nanocomposite at different temperatures (313, 323, 333, 343, 353, 363, and 373 K). The typical bands of PVA appeared along the spectra of Ag/CaTiO_3_. Where the broadening bands of PVA are due to O–H stretching vibration along the backbone of the polymer at 3278 cm^−1^, the peak at 2922 cm^−1^ is due to the stretching vibration of the C–H alkyl group, and the peak at 1702 cm^−1^ corresponds to the stretching of the PVA acetate group’s C=O bond. These bands decreased with increasing temperature and were related to the evaporation of water. The band at 1422 cm^−1^ is due to CH_2_ symmetric bending; C–H wagging vibrations could describe the peak at 1308 cm^−1^. The skeletal vibration of PVA corresponds to the peak at 820 cm^−1^^48^. It is noticed that the two characteristic peaks at 984 cm^−1^ and 1679 in the spectrum of PVA correspond to the CH_2_ asymmetric stretching^51^. The peaks at 443 and 532 cm^−1^ in the spectrum correspond to stretching vibrations of Ti–O bonds and Ca–Ti–O bonds of calcium titanate^52^. On the other hand, With the mixing of Ag/CaTiO_3_ NPs in the host matrix of PVA, notice the disappearance of the band at 1679 cm^−1^ and the intensity of the bands at 1702 and 820 cm^−1^ decreased due to the arrangement of chemical conjugation of Ag/CaTiO_3_ NPs with PVA molecules. The band at 1308 cm^−1^ disappeared, showing the decoupling between O–H and C–H vibrations related to the bonding interaction with O–H and Ag NPs.

This demonstrates that the nanoparticles interact with the PVA by the Van der Waal force, confirming the formation of PVA/Ag/CaTiO_3_ nanocomposite^53^.

It is observed that with increasing the temperature, there is a noticeable change in the intensity of the bands at 3278 cm^−1^ and 2922 cm^−1^ for the sample at 353 K. This may be due to the glass transition temperature of pure PVA, which is approximately at 354.5 K^45^.

**Figure 7:**
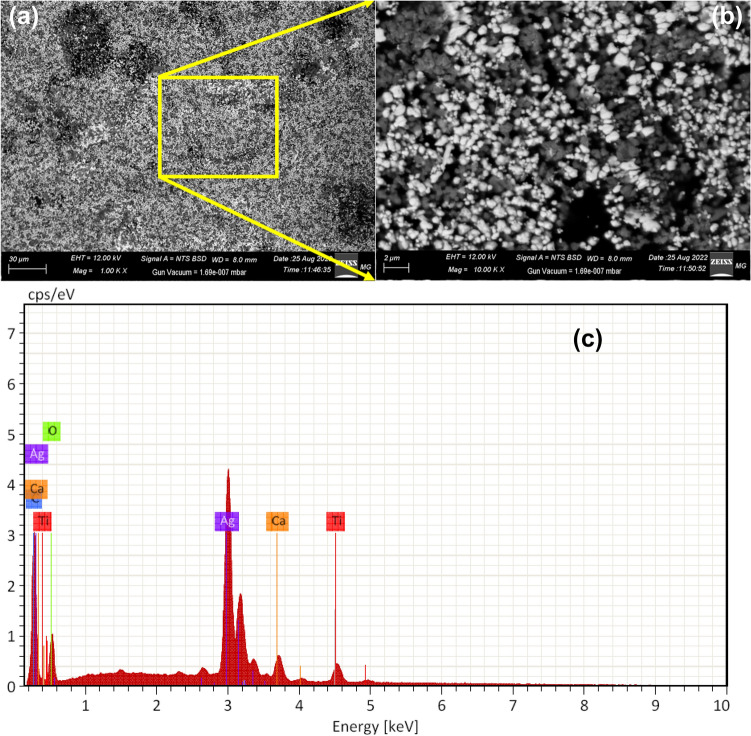
(**a**,**b**) SEM images and (**c**) EDX spectra of PVA/Ag/CaTiO_3_ nanocomposite film.

**Figure 8:**
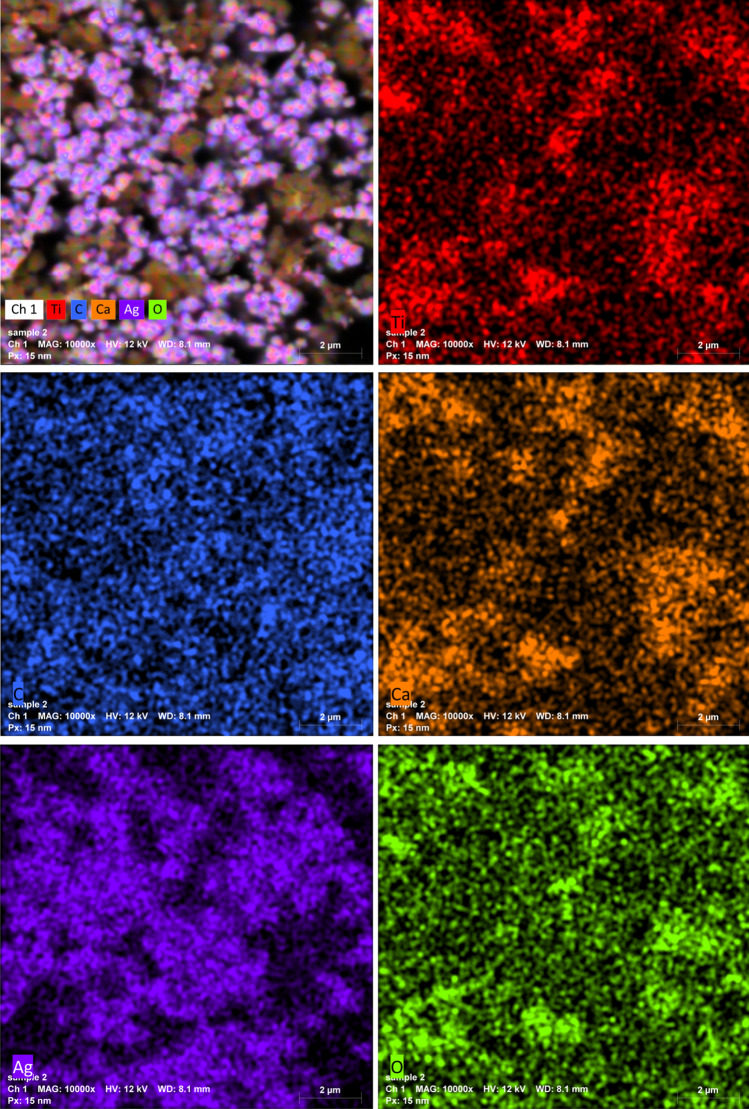
Mapping images of PVA/Ag/CaTiO_3_ nanocomposite film.

The morphology of the PVA/Ag/CaTiO_3_ nanocomposite film was assessed using SEM images. The fabricated PVA/Ag/CaTiO_3_ nanocomposite film consistently distributed Ag/CaTiO_3_ NPs in the PVA polymer matrices, as seen in the SEM images (Fig. 7a,b). EDX spectra have been employed to validate the elemental composition of the PVA/Ag/CaTiO_3_ nanocomposite film, as seen in Fig. 7c. The purity of the PVA/Ag/CaTiO_3_ nanocomposite film is proven by Fig. 7c, which shows only the elemental peaks for C, Ti, Ag, Ca, and O and no other elements peaks. The mapping images (Fig. 8) showed that Ag/CaTiO_3_ NPs were evenly distributed throughout the PVA matrix.

### Optical properties

**Figure 9:**
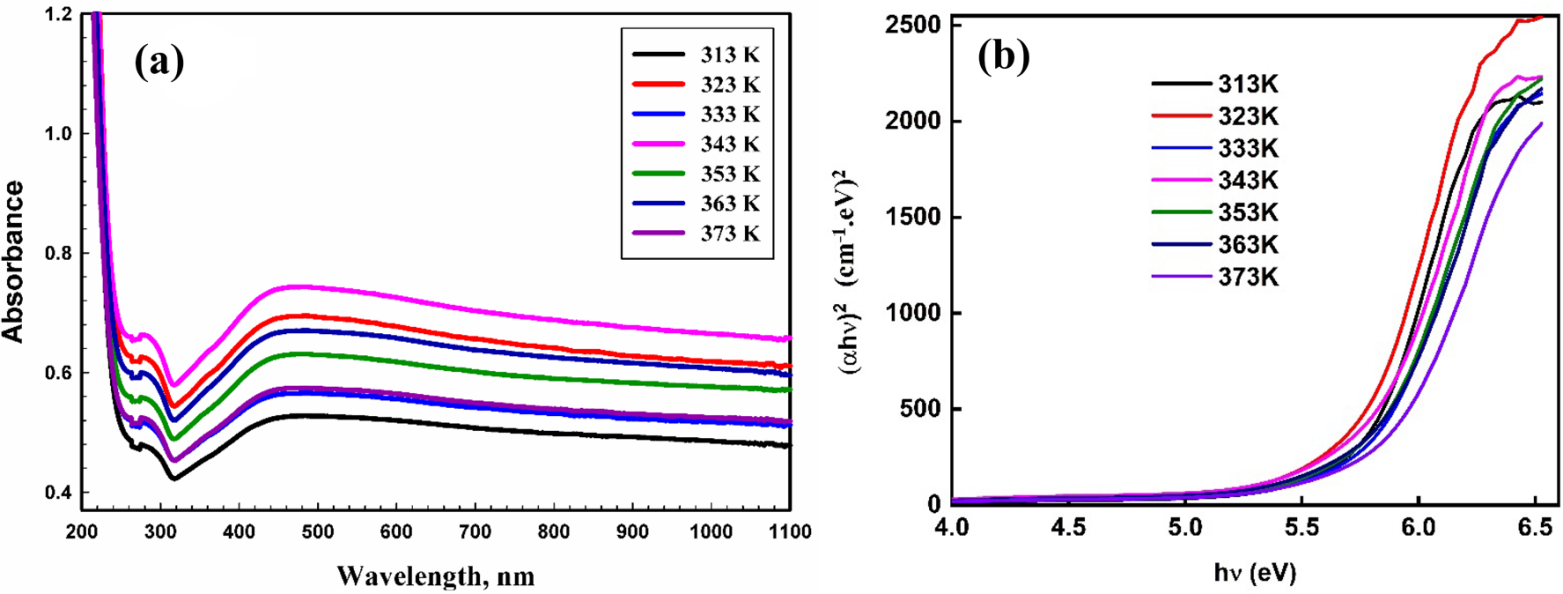
(**a**) UV–visible absorbance spectra and (**b**) (αhν)^2^ against (hν) of the PVA/Ag/CaTiO_3_ nanocomposite films.

The UV–visible absorbance spectra of the PVA/Ag/CaTiO_3_ nanocomposite films are shown in Fig. 9a. The absorption band at 288 nm corresponds to the n-π* transition^54,55^, while the apparent absorption hump at 440 nm is attributable to the surface Plasmon resonance (SPR) of Ag NP, as has been reported in our earlier work^56^. Moreover, when the temperature rises, the absorption of the polymeric films has influenced significantly. As temperatures rise, a blue shift is seen in the position of the surface Plasmon peak. Hence, the optical bandgap of materials may be expected from absorption studies, which is crucial from the perspective of technological applications. As a result, since the optical characteristics of the PVA/Ag/CaTiO_3_ nanocomposite may be directly associated with structural and electrical properties, they are critical for applications.

The absorption coefficient α(λ) is related to the optical band gap E_g_ via the following relation^57,58^:

$$\alpha h\nu ={B (h\upsilon -{E}_{g})}^{m}$$

where B signifies band tailing, hν represents photon energy, and the power m specifies the transition.

The energy gap of PVA/Ag/CaTiO_3_ nanocomposite films in direct transition at various temperatures was evaluated by plotting (αhν)^2^ against (hν), as can be seen in Fig. 9b. The direct band gap energy Eg of PVA/Ag/CaTiO_3_ nanocomposite at 313 K is found to be 5.75 eV. As the temperature increases, the direct band gap energy of PVA/Ag/CaTiO_3_ nanocomposite films increases. By increasing the temperature, the optical band gap was increased to 5.84 eV at 373 K. This increase might be due to temperature changes influencing the electronic structure of the PVA chain. In other words, an increase in Eg values induces lattice defect and enhances the degree of electronic disorder in PVA/Ag/CaTiO_3_ nanocomposite film, resulting in a loss in the crystallinity of Ag NPs. The exceptional decrease in Eg value at 323 K (5.72 eV) may be due to a change in crystallinity, as shown by XRD measurement^59^.

### Thermogravimetric analysis (TGA)

Thermal stability is essential for using PVA/Ag/CaTiO_3_ nanocomposite film  in high temperatures in optoelectronic devices. The thermal stability of PVA/Ag/CaTiO_3_ nanocomposite film was tested from 313 to 873 K at a constant rate of 10 K min^−1^ under a nitrogen atmosphere.

**Figure 10:**
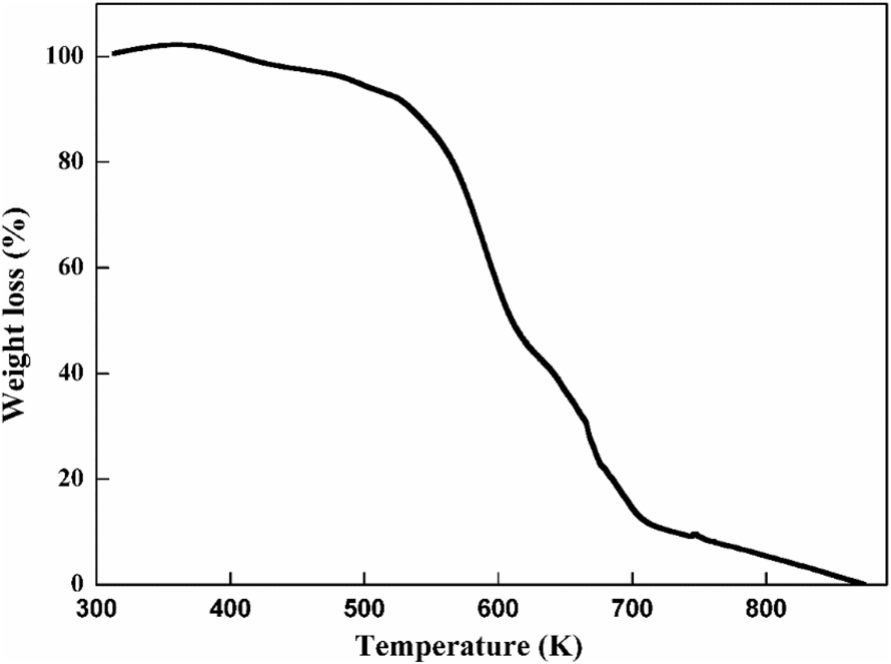
TGA curve of PVA/Ag/CaTiO_3_ nanocomposite film.

As seen in Fig. 10, the PVA/Ag/CaTiO_3_ nanocomposite film's thermo-gravimetric analysis (TGA) curve exhibits three significant weight loss areas. The first area, which occurred at a temperature ranging from 343 to 451 K, has been associated with the evaporation of water that had been slightly adsorbed, and the weight loss of the film was around 4.409%. The second area occurs between 540 and 630 K and is caused by the decomposition of the PVA polymeric matrix. At this point, the film has lost around 49.39% of its weight. The reason for the weight loss that occurred during the third stage, which was correlated with 33.38% of the film at its highest temperature of 652–682 K, was that PVA chains split into several tiny fragments. At this point, the overall percentage of losing weight is around 99.1%^60^. The thermal stability of the PVA/Ag/CaTiO_3_ nanocomposite is improved by mixing Ag/CaTiO_3_ into the PVA matrix^51,61^.

### Direct electrical conductivity

The direct electrical conductivity, denoted by the σ_dc_, is independent of the frequency and results from the free charges in the sample. The direct electrical conductivity σ_dc_ can be expressed from the relation between the resistance R of the film and its length *l* and the area* A*, as follows^62^:

$$\sigma_{dc} = L/RA$$

**Figure 11:**
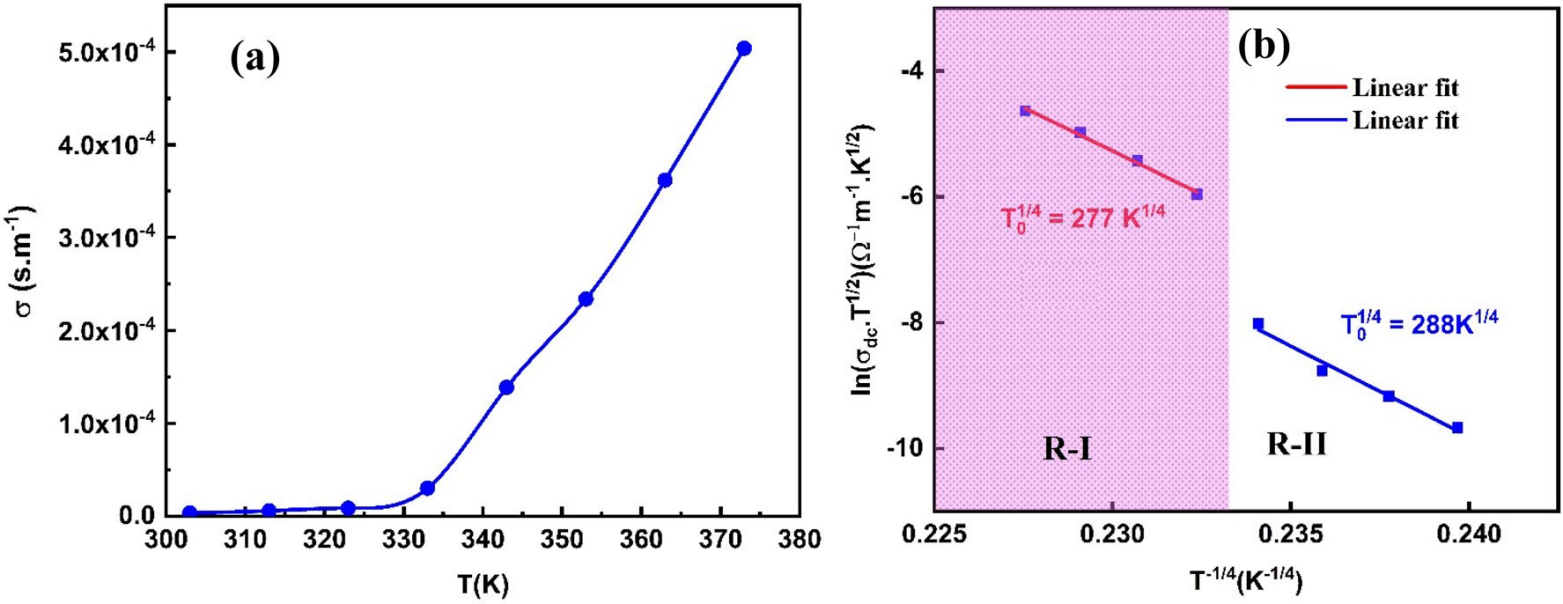
(**a**) Relation of σ_dc_ Vs. T (**b**) relation of ln (σ_dc_.T^1/2^) Vs. (T^−1/4^) for PVA/Ag/CaTiO_3_ nanocomposite film.

The dc conductivity of PVA/Ag/CaTiO_3_ nanocomposite film gradually increases with the increase in temperature, as shown in Fig. 11a. When the temperature rises, electrons from the valence band can jump to the conduction band, allowing free mobility between the two bands and improving the material's conductivity. The increased dc conductivity with the temperature reveals that the PVA/Ag/CaTiO_3_ nanocomposite film exhibits semiconductor characteristics^63^.

This work describes the conduction mechanism of σ_dc_ for PVA/Ag/CaTiO_3_ nanocomposite film in terms of a variable range hopping mechanism (VRH). The following connections provide the foundation for this explanation^64^:

$${\sigma }_{dc}{T}^{1/2}={\sigma }_{0}{e}^{{\left(\frac{-{T}_{0}}{T}\right)}^{1/4}}$$

where T_0_ is the Mott temperature.

The VRH model's validity was tested for PVA/Ag/CaTiO_3_ nanocomposite film by plotting ln (σ_dc_T^1/2^) vs. T^−1/4^ as shown in Fig. 11b. The slope of the VRH curve was used in the calculation to get the value of T_0_. As a result of our findings, we have concluded that the transport model (VRH) may be the primary low-temperature transport mechanism. The obtained value of T_0_ is 5.88 × 10^9^ K and 6.87 × 10^9^ K for PVA/Ag/CaTiO_3_ nanocomposite film in the temperature regions R-I and R-II, respectively.

### Dielectric measurements

One of the essential features of composite films is dielectric permittivity, which represents the material's propensity to polarize. It physically represents the more remarkable polarization generated in a material by an external field of specific strength. The complex permittivity describes and gives the dielectric characteristics^65^:

$$\varepsilon ={\varepsilon }_{0}({\varepsilon }_{r}-i{\varepsilon }_{i})$$

where ε_0_, ε_r_, and ε_*i*_ are the free space, real and imaginary parts of permittivity of complex dielectric constant.

**Figure 12:**
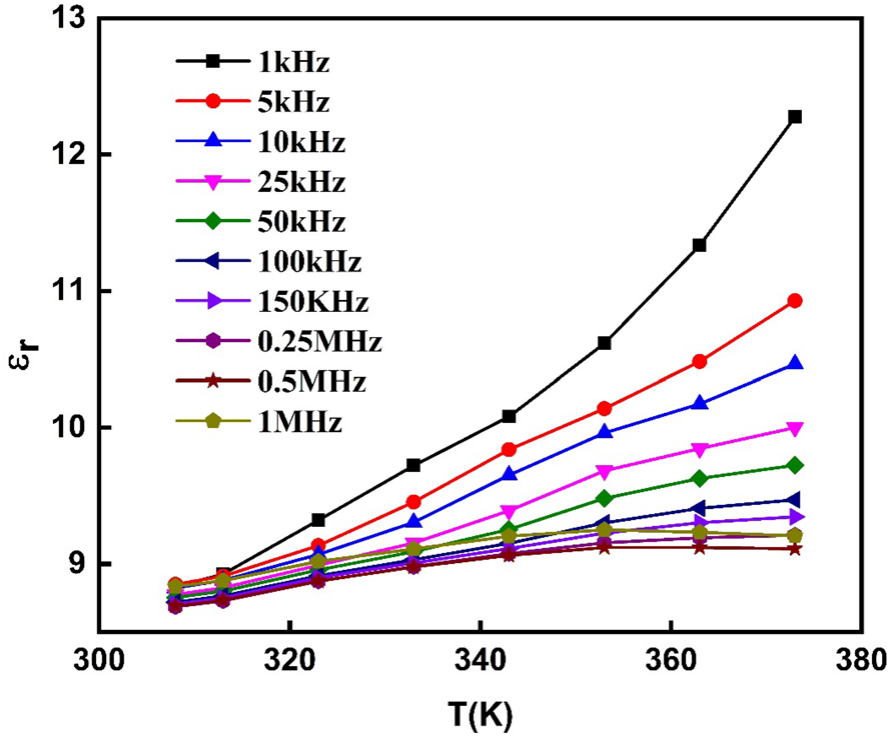
Real part of the dielectric constant (ε_r_) as a function of temperature for a range of frequencies for PVA/Ag/CaTiO_3_ nanocomposite film.

It is generally known that ε_r_ indicates the amount of electric energy stored in the material due to the applied alternating electric field. In addition, ε_r_ depicts the strength of the dipole arrangement concerning the direction of the electric field^66^. Figure 12 depicts the dependency of the dielectric constant (ε_r_) on the temperature for PVA/Ag/CaTiO_3_ nanocomposite film at different frequencies (1.0 kHz to 1.0 MHz). This figure demonstrates that the ε_r_ for PVA/Ag/CaTiO_3_ nanocomposite film rises with temperature and is decreased with frequencies. We interpret this behavior in the following manner: as the temperature of PVA/Ag/CaTiO_3_ nanocomposite film rises, the possibility of producing more charge carriers with high mobility (holes and ions) likewise rises^67^. Compared to the low temperature, the electric dipoles may efficiently align spontaneously. One possible explanation for the lower values of ε_r_ seen at high frequencies is that the charges at the interfaces are unable to realign their direction in response to the intense alternating electric field. In addition, while performing at low frequencies, the interface charges have been given the necessary time to realign themselves and participate in the ε_r_^63^.

**Figure 13:**
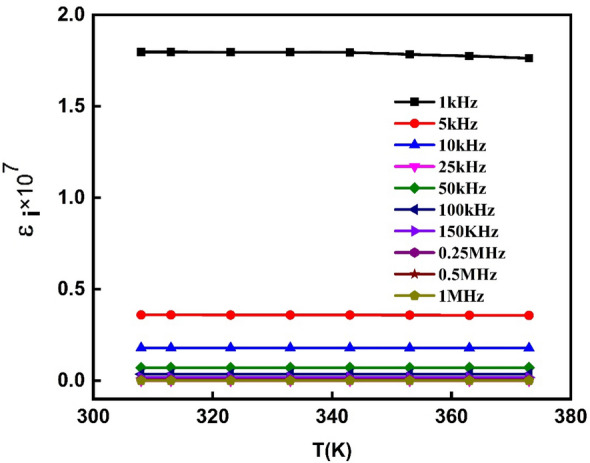
Imaginary dielectric constant (ε_i_) as a function of temperature for a range of frequencies for PVA/Ag/CaTiO_3_ nanocomposite film.

Similarly, data on the dissipated energy in the PVA/Ag/CaTiO_3_ nanocomposite film can be derived by graphing the imaginary component of the dielectric constant ε_i_ against temperature at various frequencies (1 kHz to 1.0 MHz), as illustrated in Fig. 13. It demonstrates that ε_i_ is independent of temperature but is inversely proportional to frequency, decreasing as the frequency increases. In summary, at these temperatures (313–373 K), the PVA/Ag/CaTiO_3_ nanocomposite film interface charges have appropriate mobility and contribute significantly to the dissipated energy. Regarding frequency dependency, the interface charges may rearrange themselves at low frequencies but cannot at high frequencies^63,67^.

### AC-conductivity and conduction mechanism

The AC conductivity σ_ac_ provided through various mechanisms comprises inherent charge carriers, lattice energy, defects, and impurities. The AC conductivity σ_ac_ measurements are performed to completely comprehend the conduction behavior of the parameters that may influence this mechanism of the samples. It is then considered to assess it for suitable applications^66–68^.

Furthermore, if the σ_ac_ of semiconductors refuses to obey the Arrhenius universal equation, it may be expressed as follows^69,70^:

$${\sigma }_{ac}={\sigma }_{0}{exp}^{\left(-\frac{\Delta {E}_{ac}}{{k}_{B}T}\right)}$$

where E_ac_ is the activation energy.

**Figure 14:**
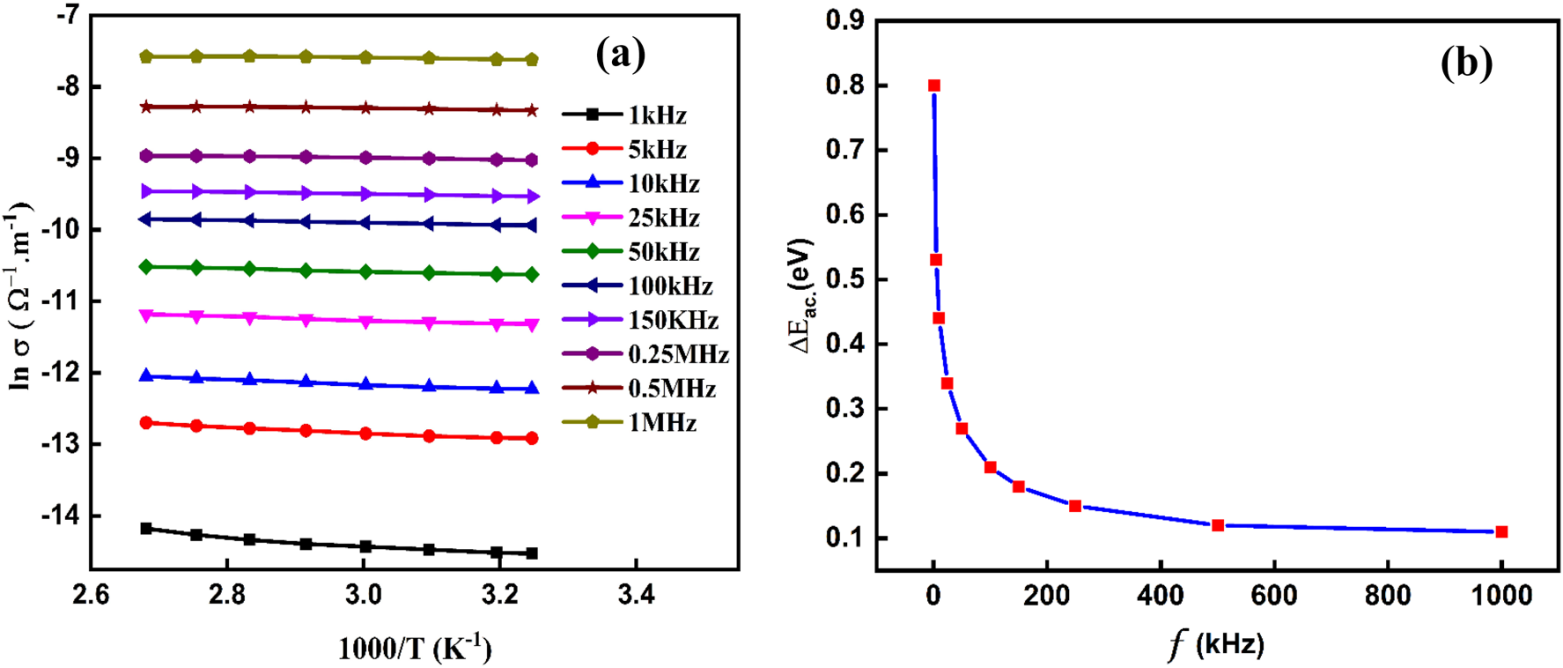
(**a**)The relationship between ln ${\sigma }_{ac}$ and 1000/T and (**b**) the activation energy E_ac_ Vs. the frequency for the PVA/Ag/CaTiO_3_ nanocomposite film.

The relationship between ln σ_ac_ and 1000/T for PVA/Ag/CaTiO_3_ nanocomposite film is shown in Fig. 14a^67^. For a given temperature, the conductivity is constant and independent of frequency in the low-frequency zone. Furthermore, Ag/CaTiO_3_ perovskite materials contain well-conductive grains surrounded by less conductive grain borders, and their activity is greater at lower frequencies. This effect leads to poor conductivity because of a modest electron jump in this location. When the frequency rises, the conductivity increases significantly. This is due to more charge carriers and more processes in the material for jumping charge carriers between consecutive sites. The phenomena then show dispersive behavior, yielding AC conduction conductivity σ_ac_^71,72^. The enhancement in conductivity with increasing temperature reveals that the conduction mechanism in the PVA/Ag/CaTiO_3_ nanocomposite film has been thermally activated. For PVA/Ag/CaTiO_3_ nanocomposite film, the activation energy reduces as the temperature increases. The activation energy E_ac_ of PVA/Ag/CaTiO_3_ nanocomposite film has been computed from the slope of linear parts for specified frequencies and is observed to be in the range 0.11–0.8 eV (see Fig. 14b).

In addition, the value of the alternating current conductivity σ_ac_ of the PVA/Ag/CaTiO_3_ nanocomposite film could potentially be described as a function of the angular frequency ω^73,74^:

$${\sigma }_{ac}=B{\omega }^{s}$$

where B would be a constant related to a given temperature and S is the frequency exponent that denotes the degree of interaction between mobile ions and lattices in the PVA/Ag/CaTiO_3_ nanocomposite film^75^.

**Figure 15:**
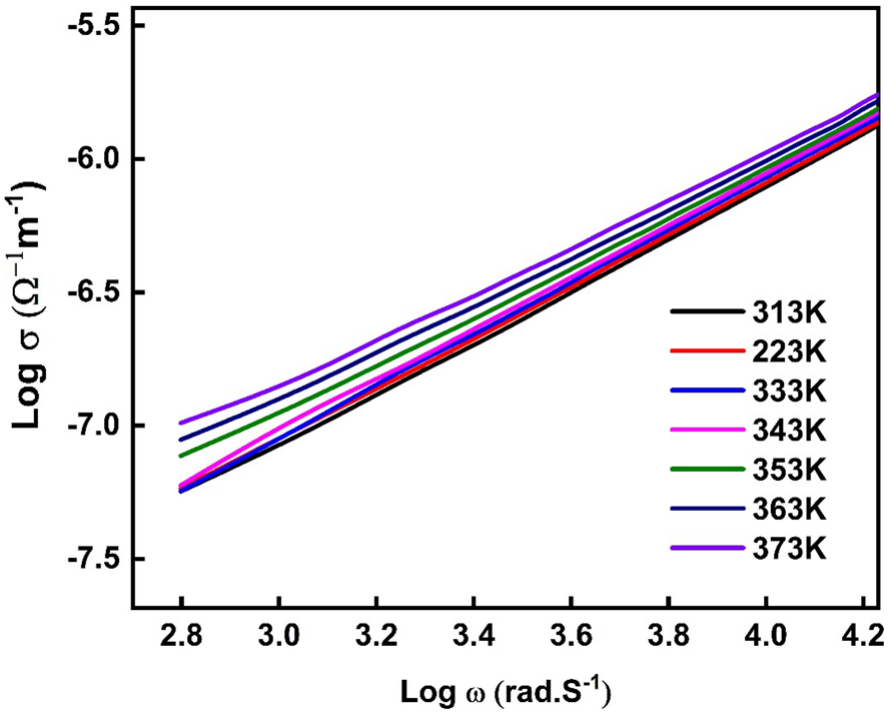
The relationship between log ${\sigma }_{ac}$ and log ω for PVA/Ag/CaTiO_3_ nanocomposite film.

The relationship between log ${\sigma }_{ac}$ and log ω for PVA/Ag/CaTiO_3_ nanocomposite film is seen in Fig. 15. The conductivity of PVA/Ag/CaTiO_3_ nanocomposite film rises sharply with frequency. Furthermore, for most semiconductors, this change will occur at a certain frequency known as the hopping frequency, which improves with increasing temperature. Our PVA/Ag/CaTiO_3_ nanocomposite film's high conductivity values suggest they could be utilized in electronic applications such as optoelectronics, electronic chips, and gas sensors.

**Figure 16:**
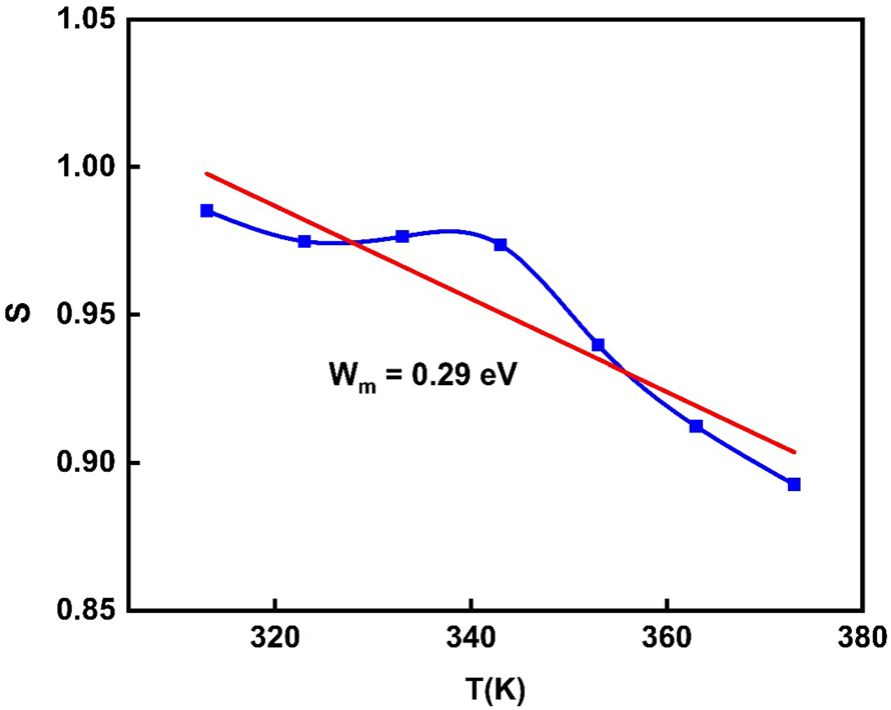
The dependency of frequency exponent, S, for PVA/Ag/CaTiO_3_ nanocomposite film on temperature.

The slopes of the straight lines in Fig. 16 at high frequencies are utilized to derive the exponent S. Figure 16 depicts the dependency of the frequency exponent, S, of PVA/Ag/CaTiO_3_ nanocomposite film on temperature. The parameter S is essential in defining the conduction mechanism in PVA/Ag/CaTiO_3_ nanocomposite film. According to Funke et al.^64^, if S is less than 1, the charge carriers suffer a transport displacement with a sudden hopping, and if S is more than 1, the species gets a located jump. In our study, PVA/Ag/CaTiO_3_ nanocomposite film has S less than 1, resulting in a transport displacement with a sudden hopping for the charge carriers. As the temperature of a PVA/Ag/CaTiO_3_ nanocomposite film rises, the exponent S decreases dramatically^67^. There is a suggestion that this behavior might be explained by the correlated barrier hopping (CBH) model. In the CBH model, the electrons react to the forces produced by an applied electrical field through jumping the potential barrier on their path from one hopping site to another. This takes place as a direct result of the electrons' potential to hop across the barrier^76^. According to the CBH model, the frequency exponent, S, can be expressed as^77^:

$$S = 1 - 6k_{B} T/W_{m}$$

where k_B_ denotes Boltzmann’s constant and W_m_ is the height of the maximum barrier (the amount of energy required to get an electron out of its ground state towards its excited state). The height of the maximum barrier W_m_ value was found of 0.29 eV.

### The dielectric modulus

Studying electric modulus formalism is a practical approach to exploring the electrical transport mechanism and learning more about the relaxation process.

The complex modulus for PVA/Ag/CaTiO_3_ nanocomposite film can be represented using an equation^78^.

$$M={M}_{r}+j{M}_{i}$$

in which M_*r*_ and M_*i*_ denoted the complex modulus's real and imaginary components.

$${M}_{r}=\frac{{\varepsilon }_{r}}{{\varepsilon }_{r}^{2}+{\varepsilon }_{i}^{2}}$$

$${M}_{i}=\frac{{\varepsilon }_{i}}{{\varepsilon }_{r}^{2}+{\varepsilon }_{i}^{2}}$$

**Figure 17:**
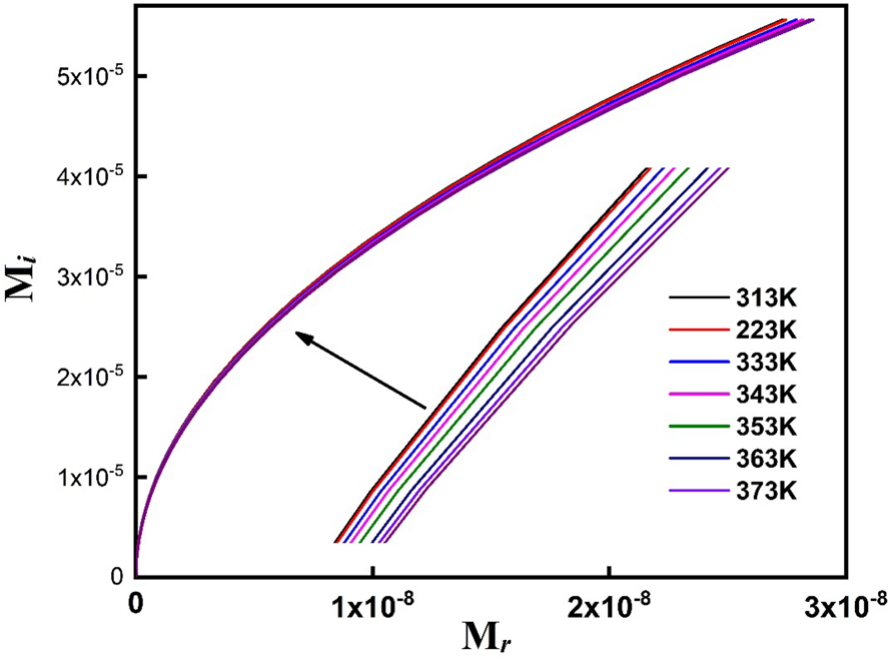
Cole–cole plots (M_i_ Vs M_r_) of PVA/Ag/CaTiO_3_ nanocomposite film.

Figure 17 depicts the cole–cole plots (M_*i*_ Vs. M_r_) for PVA/Ag/CaTiO_3_ nanocomposite film at various temperatures. For all temperatures, PVA/Ag/CaTiO_3_ nanocomposite film exhibits a semicircular arc originating from the material's grain boundary contributions, first on the low-frequency side and then on the higher-frequency side. Furthermore, each semicircular arc nearly overlaps the next with slight fluctuation for all temperatures, demonstrating an electrical relaxation development in the PVA/Ag/CaTiO_3_ nanocomposite film^78,79^.

Mohamed Bakr Mohamed and M.H. Abdel-Kader^80^ have reported that the crystallite size of ZnS increased from 4 to 10 nm as the annealing temperature rose from 300 to 500 °C. The extinction coefficient of PVA/ZnS nanocomposite improves by adding ZnS NPs at 300 °C and then reducing as the annealing temperature rises. The index of refraction increases for PVA/ZnS annealed at 300 °C and then declined as more annealed nano additives are added. Also, the direct energy gap values increase from 3.3 to 4.9 eV at 500 °C, and the indirect band gap increases from 2.3 to 4.7 eV for PVA/ZnS annealed at 500 °C.

Also, According to Sathish et al.^81^, the optical transmittance of PVA/Al_2_O_3_ thin film was approximately 80% for the as-grown film, while it boosted with annealing temperature and the band gap energy (3.74–3.78 eV) reduced with annealing temperature. The dielectric constant values ranged between 8 and 16. The obtained values for the dielectric constant are greater than those of pure PVA. The values for dielectric loss have been determined to be between 0.1 and 0.6.

## Conclusion

Herein, gamma radiation-induced synthesis of Ag/CaTiO_3_ NPs and then dispersed in a PVA matrix. The temperature-dependent structural, optical, DC electrical conductivity, and dielectric characteristics of PVA/Ag/CaTiO_3_ nanocomposite film were studied. As the temperature increased, the average crystallite sizes of CaTiO_3_ and Ag NPs decreased from 19.8 to 9.7 nm and 25 nm to 14.8, respectively. The optical band gap increased from 5.75 to 5.84 eV at 373 K. Moreover, the increase of the dc conductivity with the temperature shows that the PVA/Ag/CaTiO_3_ nanocomposite film exhibits a semiconductor behavior. The frequency exponent, S, of PVA/Ag/CaTiO_3_ nanocomposite film, gradually decreases as the temperature increases and is less than 1. Further, the maximum barrier W_m_ value is around 0.29 eV. This unique optical, DC electrical conductivity and dielectric properties of PVA/Ag/CaTiO_3_ nanocomposite film reveal that it can be used for flexible electronic devices.

## Data Availability

All data generated or analysed during this study are included in this published article.

## References

[1] Kausar, A. & Taherian, R. Electrical conductivity behavior of polymer nanocomposite with carbon nanofillers. *Electr. Conduct. Polym-Based Compos. Exp. Model. Appl. Plast. Des. Lib.* 41–72 (2018).

[2] Somesh T (2021). Polymer nanocomposites comprising PVA matrix and AgGaO_2_ nanofillers: Probing the effect of intercalation on optical and dielectric response for optoelectronic applications. Ind. J. Sci. Technol.

[3] Maajid SA, Safiulla M (2018). Investigation of electrical and thermal property of poly(vinyl alcohol)–calcium titanate nanocomposites. J. Mater. Sci. Mater. Electron..

[4] Somesh TE (2021). Polymer nanocomposites comprising PVA matrix and AgGaO_2_ nanofillers: Probing the effect of intercalation on optical and dielectric response for optoelectronic applications. Indian J. Sci. Technol..

[5] Meera K, Ramesan MT (2023). Performance of boehmite nanoparticles reinforced carboxymethyl chitosan/polyvinyl alcohol blend nanocomposites tailored through green synthesis. J. Polym. Environ..

[6] Ramesan MT (2018). Nano zinc ferrite filler incorporated polyindole/poly(vinyl alcohol) blend: Preparation, characterization, and investigation of electrical properties. Adv. Polym. Technol..

[7] Ramesan MT (2012). In situ synthesis, characterization and conductivity of copper sulphide/polypyrrole/polyvinyl alcohol blend nanocomposite. Polym.-Plast. Technol. Eng..

[8] Ramesan MT (2018). Influence of copper sulphide nanoparticles on the structural, mechanical and dielectric properties of poly(vinyl alcohol)/poly(vinyl pyrrolidone) blend nanocomposites. J. Mater. Sci. Mater. Electron..

[9] Passi M, Pal B (2022). Influence of Ag/Cu photodeposition on CaTiO_3_ photocatalytic activity for degradation of Rhodamine B dye. Korean J. Chem. Eng..

[10] Hui-Ping L, Yi-Feng D, Lin Y (2007). Anomalous optical and electronic properties of CaTiO_3_ perovskites. Commun. Theor. Phys..

[11] Shi X (2018). Synthesis of vertically aligned CaTiO_3_ nanotubes with simple hydrothermal method and its photoelectrochemical property. Nanotechnology.

[12] Yan Y (2019). Enhanced photocatalytic performance and mechanism of Au@CaTiO_3_ composites with Au nanoparticles assembled on CaTiO_3_ nanocuboids. Micromachines.

[13] Křenek T (2021). Nano and micro-forms of calcium titanate: Synthesis, properties and application. Open Ceramics.

[14] Lalan V, Mahadevan Pillai VP, Gopchandran KG (2022). Enhanced electron transfer due to rGO makes Ag–CaTiO_3_@rGO a promising plasmonic photocatalyst. J. Sci. Adv. Mater. Devices.

[15] AbdulKareem SK, Ajeel SA (2021). Effect of annealing temperatures on the structural and crystalline properties of CaTiO_3_ powder synthesized via conventional solid-state method. Mater. Today Proc..

[16] Patil B, Srinivasa R, Dharwadkar S (2007). Synthesis of CaTiO_3_ from calcium titanyl oxalate hexahydrate (CTO) as precursor employing microwave heating technique. Bull. Mater. Sci..

[17] Mallik, P. K. *et al.* Characterisation of sol-gel synthesis of phase pure CaTiO_3_ nano powders after drying. In *IOP Conference Series: Materials Science and Engineering*. (IOP Publishig, 2015).

[18] Chen T (2019). Hydrothermal synthesis of perovskite CaTiO_3_ tetragonal microrods with vertical V-type holes along the [010] direction. CrystEngComm.

[19] Passi M, Pal B (2021). A review on CaTiO_3_ photocatalyst: Activity enhancement methods and photocatalytic applications. Powder Technol..

[20] Čubová K, Čuba V (2020). Synthesis of inorganic nanoparticles by ionizing radiation—A review. Radiat. Phys. Chem..

[21] Flores-Rojas GG, López-Saucedo F, Bucio E (2020). Gamma-irradiation applied in the synthesis of metallic and organic nanoparticles: A short review. Radiat. Phys. Chem..

[22] Chen P (2007). Synthesis of silver nanoparticles by γ-ray irradiation in acetic water solution containing chitosan. Radiat. Phys. Chem..

[23] Wiguna, P. *et al.**Physicochemical properties of colloidal Ag/PVA nanoparticles synthesized by gamma irradiation*. In *Journal of Physics: Conference Series* (IOP Publishing, 2020).

[24] AbdelMaksoud MIA (2021). Gamma irradiation-assisted synthesis of PANi/Ag/MoS_2_/LiCo_0.5_Fe_2_O_4_ nanocomposite: Efficiency evaluation of photocatalytic bisphenol A degradation and microbial decontamination from wastewater. Opt. Mater..

[25] Abdel Maksoud MIA, Elsaid MAM, Abd Elkodous M (2022). Gamma radiation induced synthesis of Ag decorated NiMn_2_O_4_ nanoplates with enhanced electrochemical performance for asymmetric supercapacitor. J. Energy Storage.

[26] Abdel Maksoud MIA (2022). Gamma radiation-induced synthesis of a novel chitosan/silver/Mn-Mg ferrite nanocomposite and its impact on cadmium accumulation and translocation in brassica plant growth. Int. J. Biol. Macromol..

[27] Abdel Maksoud MIA (2022). Gamma-rays induced synthesis of Ag-decorated ZnCo_2_O_4_–MoS_2_ heterostructure as novel photocatalyst and effective antimicrobial agent for wastewater treatment application. J. Inorganic Organomet. Polym. Mater..

[28] Maksoud MA (2021). Gamma irradiation-assisted synthesis of PANi/Ag/MoS_2_/LiCo_0.5_Fe_2_O_4_ nanocomposite: Efficiency evaluation of photocatalytic bisphenol A degradation and microbial decontamination from wastewater. Opt. Mater..

[29] Ghobashy MM (2018). Radiation induced in-situ cationic polymerization of polystyrene organogel for selective absorption of cholorophenols from petrochemical wastewater. J. Environ. Manag..

[30] Bekhit M (2021). Radiation-induced synthesis of copper sulfide nanotubes with improved catalytic and antibacterial activities. Environ. Sci. Pollut. Res..

[31] Younis SA, Ghobashy MM, Samy M (2017). Development of aminated poly (glycidyl methacrylate) nanosorbent by green gamma radiation for phenol and malathion contaminated wastewater treatment. J. Environ. Chem. Eng..

[32] Sokary R (2021). A potential antibiofilm, antimicrobial and anticancer activities of chitosan capped gold nanoparticles prepared by γ-irradiation. Mater. Technol..

[33] Bekhit M (2021). Radiation-induced synthesis of copper sulfide nanotubes with improved catalytic and antibacterial activities. Environ. Sci. Pollut. Res..

[34] Lee SW, Lozano-Sanchez LM, Rodriguez-Gonzalez V (2013). Green tide deactivation with layered-structure cuboids of Ag/CaTiO_3_ under UV light. J. Hazard Mater..

[35] Maddu A, Permatasari L, Arif A (2017). Structural and dielectric properties of CaTiO_3_ synthesized utilizing Duck’s eggshell as a calcium source. J. Ceram. Process. Res..

[36] Zdorovets MV (2022). Synthesis, properties and photocatalytic activity of CaTiO_3_-based ceramics doped with lanthanum. Nanomaterials.

[37] Krumm, S. *An interactive Windows program for profile fitting and size/strain analysis*. In *Materials Science Forum* (Trans Tech Publ, 1996).

[38] Dou P (2015). One-step microwave-assisted synthesis of Ag/ZnO/graphene nanocomposites with enhanced photocatalytic activity. J. Photochem. Photobiol. A.

[39] Kottappara R, Palantavida S, Vijayan BK (2020). A facile synthesis of Cu–CuO–Ag nanocomposite and their hydrogenation reduction of p-nitrophenol. SN Appl. Sci..

[40] Gayathri, S. *et al.**Investigation of physicochemical properties of Ag doped ZnO nanoparticles prepared by chemical route.* (2015).

[41] Ahmad K, Kumar P, Mobin SM (2020). Hydrothermally grown novel pyramids of the CaTiO_3_ perovskite as an efficient electrode modifier for sensing applications. Mater. Adv..

[42] Kumar S (2019). Bio-based (chitosan/PVA/ZnO) nanocomposites film: Thermally stable and photoluminescence material for removal of organic dye. Carbohydr. Polym..

[43] Prashanth K (2016). Solution combustion synthesis of Cr_2_O_3_ nanoparticles and derived PVA/Cr_2_O_3_ nanocomposites-positron annihilation spectroscopic study. Mater. Today Proc..

[44] Ray SS, Bousmina M (2005). Biodegradable polymers and their layered silicate nanocomposites: In greening the 21st century materials world. Prog. Mater Sci..

[45] Dong C (2021). Insight into glass transition temperature and mechanical properties of PVA/TRIS functionalized graphene oxide composites by molecular dynamics simulation. Mater. Des..

[46] Singh, M. K. & Singh, A. Thermal characterization of materials using differential scanning calorimeter. *J. Charact. Polym. Fibres* 201–222 (2022).

[47] Domínguez J (2018). Rheology and curing process of thermosets. Thermosets.

[48] Shikinaka K (2018). Tuneable shape-memory properties of composites based on nanoparticulated plant biomass, lignin, and poly (ethylene carbonate). Soft Matter.

[49] Sasidharan S (2020). Perovskite titanates at the nanoscale: Tunable luminescence by energy transfer and enhanced emission with Li^+^ co-doping. J. Solid State Chem..

[50] Chahal RP (2012). γ-Irradiated PVA/Ag nanocomposite films: Materials for optical applications. J. Alloy. Compd..

[51] Xiong Y (2009). Performance of organic–inorganic hybrid anion-exchange membranes for alkaline direct methanol fuel cells. J. Power Sources.

[52] Liu J (2015). Synthesis of MoS_2_/SrTiO_3_ composite materials for enhanced photocatalytic activity under UV irradiation. J. Mater. Chem. A.

[53] Kashyap S, Pratihar SK, Behera SK (2016). Strong and ductile graphene oxide reinforced PVA nanocomposites. J. Alloy. Compd..

[54] Aziz SB (2019). Structural and optical characteristics of PVA:C-dot composites: Tuning the absorption of ultra violet (UV) region. Nanomaterials.

[55] Zidan HM (2019). Characterization and some physical studies of PVA/PVP filled with MWCNTs. J. Market. Res..

[56] Abdel Maksoud MIA (2021). Effect of gamma irradiation on the free-standing polyvinyl alcohol/chitosan/Ag nanocomposite films: Insights on the structure, optical, and dispersion properties. Appl. Phys. A.

[57] Sallam OI (2022). Enhanced linear and nonlinear optical properties of erbium/ytterbium lead phosphate glass by gamma irradiation for optoelectronics applications. Appl. Phys. A.

[58] Abou Hussein EM (2021). Unveiling the gamma irradiation effects on linear and nonlinear optical properties of CeO_2_–Na_2_O–SrO–B_2_O_3_ glass. Opt. Mater..

[59] El Askary A (2022). Optical, thermal, and electrical conductivity strength of ternary CMC/PVA/Er_2_O_3_ NPs nanocomposite fabricated via pulsed laser ablation. Phys. B.

[60] Yang C-C (2007). Synthesis and characterization of the cross-linked PVA/TiO_2_ composite polymer membrane for alkaline DMFC. J. Membr. Sci..

[61] Heydari M (2013). Effect of cross-linking time on the thermal and mechanical properties and pervaporation performance of poly (vinyl alcohol) membrane cross-linked with fumaric acid used for dehydration of isopropanol. J. Appl. Polym. Sci..

[62] Webster JG (2003). Electrical Measurement, Signal Processing, and Displays.

[63] El-ghandour A (2019). Temperature and frequency dependence outline of DC electrical conductivity, dielectric constants, and AC electrical conductivity in nanostructured TlInS_2_ thin films. Physica E.

[64] Elliott SR (1987). A.C. conduction in amorphous chalcogenide and pnictide semiconductors. Adv. Phys..

[65] Morad I (2020). Effect of the biphase TiO_2_ nanoparticles on the dielectric and polaronic transport properties of PVA nanocomposite: Structure analysis and conduction mechanism. Vacuum.

[66] Sankarappa T (2008). AC conductivity and dielectric studies in V_2_O_5_–TeO_2_ and V_2_O_5_–CoO–TeO_2_ glasses. J. Mol. Struct..

[67] El-Nahass MM (2018). Structural investigation, thermal analysis and AC conduction mechanism of thermally evaporated alizarin red S thin films. Optik.

[68] El-Menyawy EM, Zeyada HM, El-Nahass MM (2010). AC conductivity and dielectric properties of 2-(2,3-dihydro-1,5-dimethyl-3-oxo-2-phenyl-1H-pyrazol-4-ylimino)-2-(4-nitrophenyl)acetonitrile thin films. Solid State Sci..

[69] Macedo, P. B. & Moynihan, C. T. *The Role of Ionic Diffusion in Polarisation in Vitreous Ionic Conductors* (1972).

[70] Day DR (1985). The role of boundary layer capacitance at blocking electrodes in the interpretation of dielectric cure data in adhesives. J. Adhes..

[71] Gharbi S (2022). Influence of calcium substitution on structural, morphological and electrical conductivity properties of La_1-x_Ca_x_Ni_0.5_Ti_0.5_O_3_ (x = 0.0, x = 0.2) compounds for energy storage devices. Inorg. Chem. Commun..

[72] Sharma J (2015). Study of dielectric properties of nanocrystalline cobalt ferrite upto microwave frequencies. Macromol. Symp..

[73] El-Ghamaz NA (2013). Conducting polymers. VI. Effect of doping with iodine on the dielectrical and electrical conduction properties of polyacrylonitrile. Solid State Sci..

[74] Zeyada HM, El-Nahass MM (2008). Electrical properties and dielectric relaxation of thermally evaporated zinc phthalocyanine thin films. Appl. Surf. Sci..

[75] Morii K (1995). Dielectric relaxation in amorphous thin films of SrTiO_3_ at elevated temperatures. J. Appl. Phys..

[76] Long A (1982). Frequency-dependent loss in amorphous semiconductors. Adv. Phys..

[77] Alosabi A (2022). Electrical conduction mechanism and dielectric relaxation of bulk disodium phthalocyanine. Phys. Scr..

[78] Gajula GR (2020). An investigation on the conductivity, electric modulus and scaling behavior of electric modulus of barium titanate-lithium ferrite composite doped with Nb, Gd and Sm. Mater. Chem. Phys..

[79] Rayssi C (2018). Frequency and temperature-dependence of dielectric permittivity and electric modulus studies of the solid solution Ca_0.85_Er_0.1_Ti_1-x_Co_4x_/3O_3_ (0 ≤ x ≤ 01). RSC Adv..

[80] Mohamed MB, Abdel-Kader MH (2020). Effect of annealed ZnS nanoparticles on the structural and optical properties of PVA polymer nanocomposite. Mater. Chem. Phys..

[81] Sugumaran S, Bellan CS, Nadimuthu M (2015). Characterization of composite PVA–Al_2_O_3_ thin films prepared by dip coating method. Iran. Polym. J..

